# Improving Estimation of Winter Wheat Nitrogen Status Using Random Forest by Integrating Multi-Source Data Across Different Agro-Ecological Zones

**DOI:** 10.3389/fpls.2022.890892

**Published:** 2022-06-10

**Authors:** Yue Li, Yuxin Miao, Jing Zhang, Davide Cammarano, Songyang Li, Xiaojun Liu, Yongchao Tian, Yan Zhu, Weixing Cao, Qiang Cao

**Affiliations:** ^1^MARA Key Laboratory for Crop System Analysis and Decision Making, Jiangsu Key Laboratory for Information Agriculture, National Engineering and Technology Center for Information Agriculture, MOE Engineering and Research Center for Smart Agriculture, Collaborative Innovation Center for Modern Crop Production Co-sponsored by Province and Ministry, Nanjing Agricultural University, Nanjing, China; ^2^Precision Agriculture Center, Department of Soil, Water and Climate, University of Minnesota, St. Paul, MN, United States; ^3^Department of Crop and Soil Sciences, University of Georgia, Tifton, GA, United States; ^4^Department of Agroecology, Aarhus University, Tjele, Denmark; ^5^Department of Land Surveying and Geo-Informatics, The Hong Kong Polytechnic University, Kowloon, China

**Keywords:** precision nitrogen management, machine learning, environmental variables, management practices, variable selection, nitrogen nutrition index

## Abstract

Timely and accurate estimation of plant nitrogen (N) status is crucial to the successful implementation of precision N management. It has been a great challenge to non-destructively estimate plant N status across different agro-ecological zones (AZs). The objective of this study was to use random forest regression (RFR) models together with multi-source data to improve the estimation of winter wheat (*Triticum aestivum* L.) N status across two AZs. Fifteen site-year plot and farmers' field experiments involving different N rates and 19 cultivars were conducted in two AZs from 2015 to 2020. The results indicated that RFR models integrating climatic and management factors with vegetation index (R^2^ = 0.72–0.86) outperformed the models by only using the vegetation index (R^2^ = 0.36–0.68) and performed well across AZs. The Pearson correlation coefficient-based variables selection strategy worked well to select 6–7 key variables for developing RFR models that could achieve similar performance as models using full variables. The contributions of climatic and management factors to N status estimation varied with AZs and N status indicators. In higher-latitude areas, climatic factors were more important to N status estimation, especially water-related factors. The addition of climatic factors significantly improved the performance of the RFR models for N nutrition index estimation. Climatic factors were important for the estimation of the aboveground biomass, while management variables were more important to N status estimation in lower-latitude areas. It is concluded that integrating multi-source data using RFR models can significantly improve the estimation of winter wheat N status indicators across AZs compared to models only using one vegetation index. However, more studies are needed to develop unmanned aerial vehicles and satellite remote sensing-based machine learning models incorporating multi-source data for more efficient monitoring of crop N status under more diverse soil, climatic, and management conditions across large regions.

## Introduction

Winter wheat (*Triticum aestivum* L.), a major staple food crop, is vital to global food security and sustainable agriculture. A great challenge in winter wheat production is to optimize nitrogen (N) management to achieve high crop yield and high N use efficiency (NUE) under different soil landscapes and weather conditions across large regions. Precision N management (PNM) has the potential to overcome this challenge by matching N application and crop demand (Cao et al., 2015; Cammarano et al., 2021). Timely and accurate estimation of plant N status is crucial to the successful implementation of PNM (Li et al., 2022). The N nutrition index (NNI) is considered as a reliable N status indicator, which can be calculated as the ratio of the actual N concentration to the critical N concentration (N_c_) (Lemaire et al., 2008).

Instead of destructive sampling and laborious analysis, active canopy sensors have been used for the estimation of crop N status based on spectral reflection and absorption properties of the crop canopy. These active canopy sensors have their own light sources and are not affected by environmental light conditions (Cao et al., 2017a). Vegetation indices (VIs) derived from these sensors and simple linear regression (SLR) have been commonly used for crop estimation (Cao et al., 2015; Bonfil, 2017). However, SLR models generally do not work well when applied in other areas or years (Munoz-Huerta et al., 2013). It is hypothesized that adding climatic and management factors can provide complementary information and improve the estimation of crop N status compared to approaches that only use VIs. Though prior studies have investigated the contributions of climatic and remote sensing data on wheat yield prediction at regional scales (Cai et al., 2019), there were limited studies that explored their contributions to the estimation of N status. Besides, diverse external factors, such as climatic and management factors, show different patterns owing to latitude and longitude.

Stepwise multiple linear regression has been commonly used for plant N status estimation using crop sensing data alone or together with other ancillary data (Miao et al., 2009; Dong et al., 2021). Recently, machine learning (ML) algorithms have been increasingly employed to combine multiple VIs or VIs with genetics, environmental, and management information to predict crop N status due to their capabilities to deal with both linear and nonlinear relationships (Zha et al., 2020; Li et al., 2022). Random forest, developed by Breiman (2001), is a representative ML algorithm with a good performance by averaging an ensemble of trees without overfitting problems (Rhee and Im, 2017; Zhang et al., 2019). It has been widely used for regression and classification applications (Wang et al., 2016), including crop N status prediction (Han et al., 2019; Lu et al., 2019; Zha et al., 2020; Li et al., 2022).

Studies developing models using crop sensing and multi-source ancillary data with ML algorithms for winter wheat N status prediction across different agro-ecological zones (AZs) are still limited. Therefore, the objective of this study was to evaluate the performance of random forest regression (RFR) models for winter wheat N status prediction across two AZs using active crop sensor data together with climatic and management information.

## Materials and Methods

### Study Area and Experiment Design

**Figure 1:**
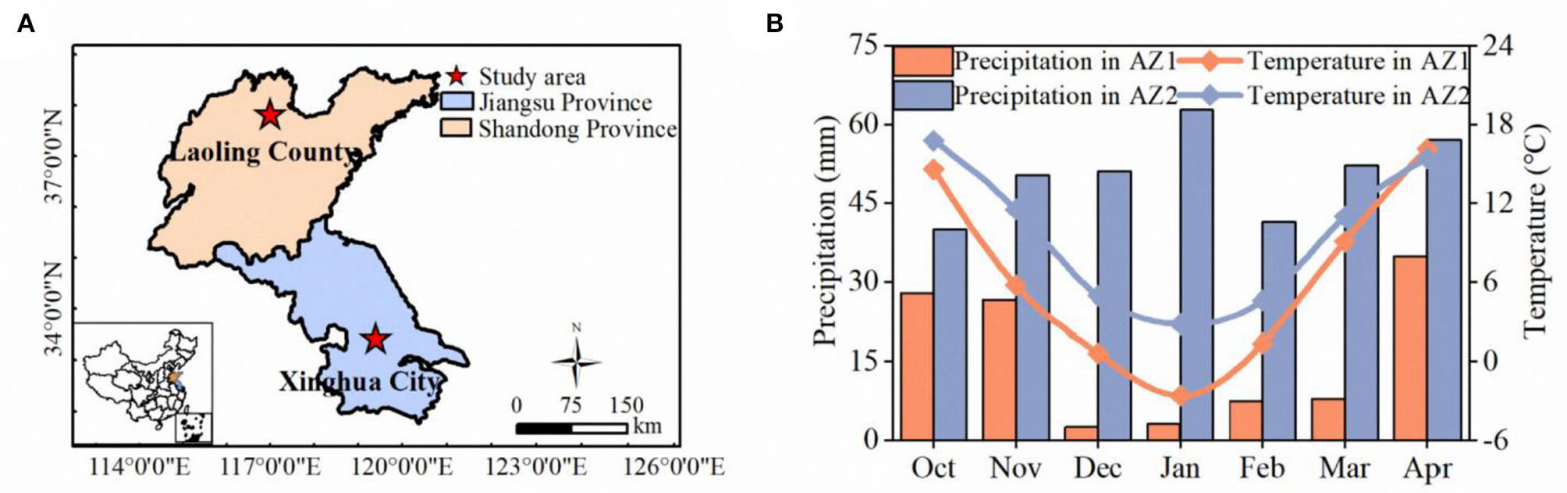
Locations of the study region **(A)**, mean precipitation (mm) and temperature (°C) of agro-ecological zone1 (AZ1, from 2015 to 2018) and agro-ecological zone 2 (AZ2, from 2017 to 2020) from sowing to sensing date **(B)**.

Fifteen site-year winter wheat field experiments were conducted in agro-ecological zone 1 (AZ1, 37°43'N and 117°13'E, in Laoling County of Shandong Province) and agro-ecological zone 2 (AZ2, 33°05'N and 119°53'E, in Xinghua City of Jiangsu Province) (Figure 1A), with different patterns of precipitation distribution and monthly average temperature (Figure 1B). The soil types of AZ1 and AZ2 were sandy loam soil and loam soil, respectively.

The design includes seven plot experiments (Exp. 1–7) and eight farmers' field experiments (Exp. 8–15) involving different N rates, sowing dates (SD), and seeding rates (SR). The N fertilizers of Exp. 1–3 were applied with 40% as basal N before sowing, except for farmer's management (FM), which applied 50% as basal N. The remaining N was applied at the stem elongation (SE) stage. The N fertilizers of Exp. 4–5 were applied at the rate of 50% of total N rates before sowing and 50% at the SE stage, respectively. The differences between Exp. 6–7 and Exp. 4–5 are listed in Table 1. With a randomized complete block design, the treatments of plot experiments were carried out with three repetitions. In addition to plot experiments, eight farmers' field experiments (Exp. 8-15) were conducted across the Nanxia village in Shandong Province to compare different N management strategies. In each farmers' field experiment, there were three treatments: FM, regional optimum N management (RONM), and PNM. RONM, integrating high-yielding and high-NUE, was managed with 81 kg N ha^−1^ as basal N and 138 kg N ha^−1^ as topdressing N (Zhou et al., 2017). For PNM, the basal N rate was the same as RONM, and topdressing N was applied according to an active canopy sensors-based algorithm (Cao et al., 2017b). Other managements were the same as RONM. Irrigation was done two times at the sowing and SE stages in AZ1 and once after sowing in AZ2. All N source was urea. For all plots, wheat was not limited by phosphate and potash fertilizers and was kept free of pests and diseases during the growing seasons.

**Table 1:** Basic information of the field experimental design.

**Sites**	**Experiments and years**	**Cultivars**	**N rates**	**P rates**	**K rates**	**Seeding rates**	**Sowing dates**	**Sensing dates**
			**(kg ha^−1^)**					
AZ1	Exp.1 (2015)	JM22, LY502	0, 120, 180, 240, 300	120	75	187.4	Oct.30^th^	Apr.17^th^
	Exp.2 (2016)	JM22, LX77	0, 120, 180, 240, 300, FM				Oct.19^th^	Apr.2^nd^
	Exp.3 (2017)	JM22, SN29					Oct.20^th^	Apr.12^th^
AZ2	Exp.4 (2017); Exp.5 (2018)	ZM12, YM23, NM13	0, 90, 180, 270, 360	105	135	133.8	Nov.8^th^; Nov.1^st^	Apr.1^st^; Mar.16^th^
AZ2	Exp.6 (2018); Exp.7 (2019)	YM23	0, 180, 240, 300	105	135	103.5, 155.3, 207.0; 112.3, 168.4, 224.6	Nov.4^th^, Nov.24^th^, Dec.1^st^; Nov.1^st^, Nov.15^th^, Dec.24^th^	Mar.18^th^, 23^th^, Apr.4^th^; Mar.14^th^, 14^th^, Apr.1^st^
AZ1		JM22	FM: 280.5;	120;	36;	300;	Oct.dd^#;^	Mar.26^th^
			RONM: 219.0;	112	27	165	Oct.dd^#;^	
	Exp.8 (2016); Exp.9 (2016); Exp.10 (2016); Exp.11 (2016); Exp.12 (2016); Exp.13 (2016); Exp.14 (2016); Exp.15 (2016)		PNM1: 199.9; PNM2: 188.4; PNM3: 208.2; PNM4: 205.0; PNM5: 213.2; PNM6: 209.4; PNM7: 200.2; PNM8: 182.5				Oct.5^th;^ Oct.2^nd;^ Oct.13^th;^ Oct.11^th;^ Oct.12^th;^ Oct.13^th;^ Oct.11^th;^ Oct.12^th^	

*FM, RONM, and PNM1-8 refer to farmers' management, regional optimum N management, and precision N management, according to Cao et al. (2017b), respectively. JM22, LY502, LX77, SN29, ZM12, YM23, and NM13 refer to Jimai22, Luyuan502, Liangxing77, Shannong29, Zhenmai12, Yangmai23 and Ningmai13, respectively*.

*AZ1 and AZ2 mean agro-ecological zone 1 and agro-ecological zone 2*.

*dd^#^ means sowing date of FM and RONM varied according to the eight farmers while being the same with PNM, respectively*.

### Data Acquisition and Calculation

#### VIs Datasets

The portable active sensor RapidSCAN CS-45 (Holland Scientific Inc., Lincoln, Nebraska, USA) was used to collect canopy reflectance and VIs data, including the default normalized difference vegetation index (NDVI) and the normalized difference red edge (NDRE). It was carried by hand at a constant speed to collect sensor readings at a sensor-to-canopy distance of approximately 0.7–0.9 m. The average values from three rows in each plot were then calculated to represent the plot. The NDRE was selected to monitor the N status to avoid the potential saturation effect of NDVI under the high aboveground biomass (AGB) and plant N uptake (PNU) conditions (Li et al., 2010). The NDRE was calculated as follows:


(1) $$\begin{array}{l} \text{NDRE }=\;\left(\text{NI}{\text{R}}_{780}\;-\text{ R}{\text{E}}_{730}\right)\;/\;\left(\text{NI}{\text{R}}_{780}\;+\text{ R}{\text{E}}_{730}\right) \end{array}$$

where *NIR*_780_ and *RE*_730_ refer to near-infrared (780nm) and red-edge (730nm) waveband reflectance, respectively.

#### Agronomic Datasets

The plants were sampled right after sensing data was collected at the SE stage (Table 1), which is the start of rapid crop growth and N uptake and the stage important for making in-season N recommendations (Meng et al., 2013).

In AZ1, plant samples were collected from randomly selected 1 m by 0.3 m areas in each plot and rinsed with water, and the roots were removed. The plant samples were placed in an oven at 105°C for 30 min and dried at 80°C to constant weight for the determination of AGB, which was converted to the unit of t ha^−1^ based on the row spacing. The sub-samples were ground to pass through a 1 mm sieve in a Wiley mill, and the plant N concentration was determined using the Kjeldahl method (Nelson and Sommers, 1973). The PNU was calculated by multiplying AGB by plant N concentration (Lu et al., 2017). According to the definition of NNI, N_c_ was calculated from the critical N dilution curves developed by Yue et al. (2012) in AZ1.


(2) $$\begin{array}{l} {\text{N}}_{\text{c}}\;=\;4.15\;\times \text{ AG}{\text{B}}^{-0.38} \end{array}$$

where AGB refers to the aboveground biomass (t ha^−1^).

Compared to AZ1, twenty plants in each plot were sampled randomly and averaged as the mean value of the plot in AZ2 experiments. The critical N dilution curve developed by Zhao et al. (2012) was used to calculate the N_c_.


(3) $$\begin{array}{l} {\text{N}}_{\text{c}}\;=\;4.33\;\times \text{ AG}{\text{B}}^{-0.45} \end{array}$$

where AGB refers to the aboveground biomass (t ha^−1^).

#### Climatic Datasets

The primary climatic variables of AZ1 and AZ2 were obtained from the China Meteorological Data Service Center (http://data.cma.cn) and the Xinghua weather station, respectively. The climatic variables from the date of sowing to sensing included seasonal total precipitation (PPT), seasonal maximum, minimum, and mean temperature (T_max_, T_min_, T_mean_), growing degree days (GDD) (Wang et al., 2014), abundant and well-distributed rainfall (AWDR) (Bean et al., 2018), shannon diversity index (SDI), relative humidity (HU), and solar radiation (RAD) calculated using the Hybrid-Maize model (Yang et al., 2004). T_max_ and T_min_ were determined as the maximum value of the daily maximum temperature (D_max_) and the minimum value of the daily minimum temperature (D_min_), respectively. GDD, SDI, and AWDR were calculated as follows:


(4) $$\begin{array}{l} \text{GDD }=\;\sum \;\left(\left({\text{D}}_{\max}\;+\;{\text{D}}_{\min}\right)\;/\;2\;-\;{\text{T}}_{\text{base}}\right) \end{array}$$

(5) $$\begin{array}{l} \text{SDI }=\;\sum \;\left(\left(-{\text{p}}_{\text{i}}\;\times \text{ ln}\left({\text{p}}_{\text{i}}\right)\right)\;/\text{ ln}\left(\text{d}\right)\right) \end{array}$$

(6) $$\begin{array}{l} \text{AWDR }=\text{ PPT }\times \text{ SDI} \end{array}$$

where *D*_*max*_= daily maximum temperature (up to 30 °C), *D*_*min*_ = daily minimum temperature, and *T*_*base*_ = base temperature (0 °C). *p*_*i*_ is the ratio of daily precipitation to *PPT*, and *d* is the days from sowing to sensing.

#### Management Datasets

Management practices included SD, SR, and basal N (BN) (shown in Table 1).

### Data Analysis

**Figure 2:**
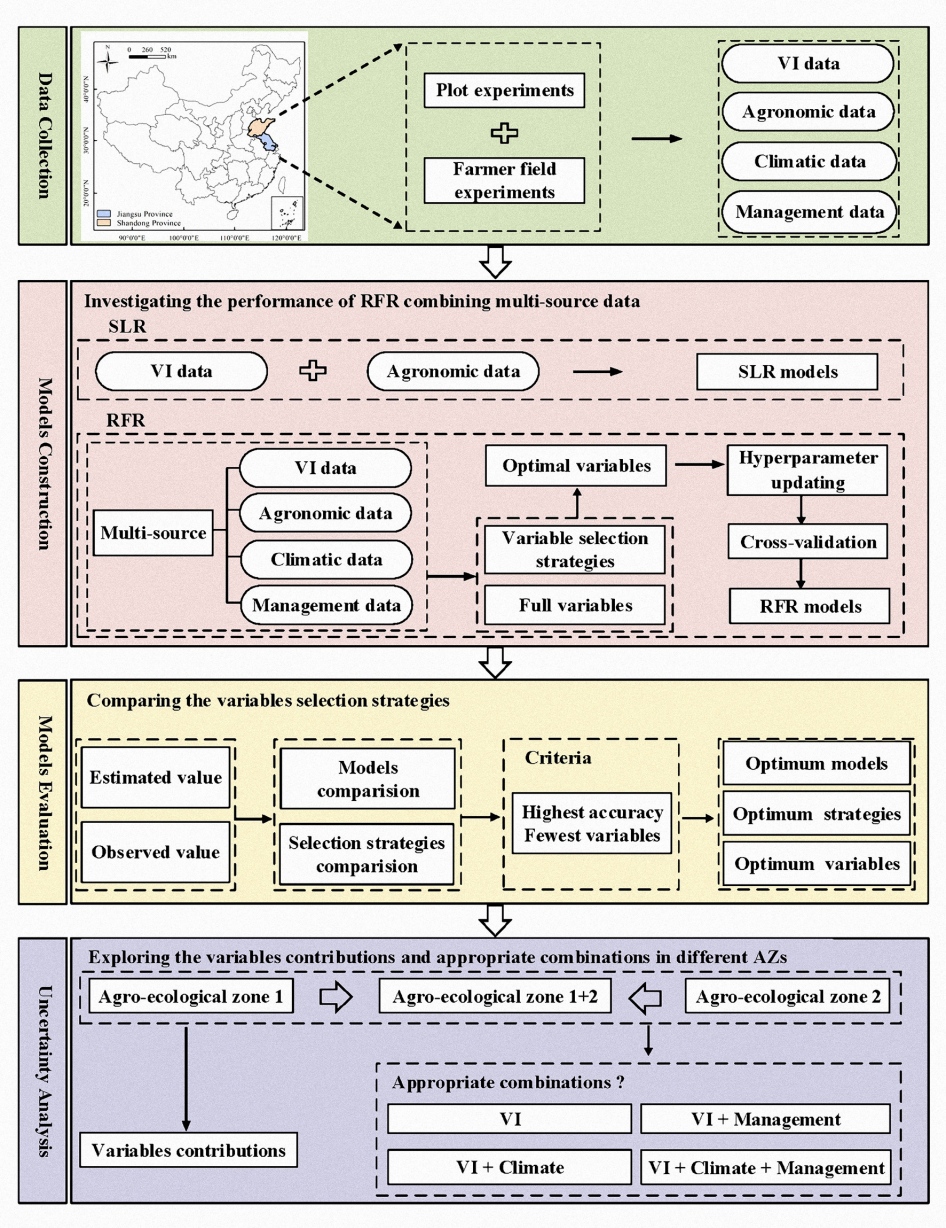
Workflow of this study.

The data were analyzed using the following steps: (1) establishing the SLR models only using NDRE; (2) identifying optimal variables based on variable selection strategies; (3) training and validating the RFR models using selected variables by updating hyperparameters and cross-validation; (4) comparing and evaluating the robustness of different models; (5) determining the variable contributions in different AZs, and (6) exploring the effective combinations of multi-source data for N status estimation (Figure 2).

#### Strategies of Variable Selection

Two proposed variable selection strategies were evaluated in this study. The first strategy was the Pearson correlation coefficient (PCC), a common method to identify the variables with the highest statistical significance. In the previous studies, it was commonly adopted to quantify the degrees of correlation between variables (Li et al., 2019; Hamrani et al., 2020). Taking AGB, PNU, and NNI as dependent variables, climatic and management data as candidate independent variables, and NDRE as a fixed independent variable, the PCC was used to select the optimal variables from the candidate independent variables within the same group to remove the compound effects. It should be emphasized that the climatic data were divided into two sub-groups: temperature-related variables (GDD, RAD, T_mean_, T_min_, and T_max_), and water-related variables (PPT, SDI, AWDR, and HU). Then, two candidate independent variables would be selected from the temperature-related, water-related group, and management groups, respectively. The selection process included the following steps: (1) selecting the variables with the maximum absolute correlation with dependent variables in each group as the first group of candidate independent variables; (2) selecting the second candidate independent variables that had smaller correlation coefficients (<0.5 threshold in this study) with the first group of candidate independent variables and the maximum correlation with the dependent variables (Wang et al., 2020).

For the second strategy, the variable importance ranked by the RFR (VIRRFR) was also adopted to select the variables (Zhu et al., 2021). It was performed specifically by (1) ranking the variable importance by the RFR; (2) adding the variables iteratively into the RFR from highest to lowest according to the importance score order until all independent variables were included; (3) determining optimal variables corresponding to the models' coefficients of determination (R^2^), root mean square error (RMSE), and relative error (RE) based on the cross-validation. The above processes were implemented to explore optimal variables for estimating AGB, PNU, and NNI across AZ1, AZ2, and the two AZs (AZ1+2), respectively.


(7) $$\begin{array}{l} \begin{array}{c} \text{RMSE = ((}\sum_{\text{n=1}}^{\text{n}}{{\text{(E}}_{\text{i}}{\text{ - O}}_{\text{i}}\text{)}}^{\text{2}}{\text{) / n)}}^{\text{1/2}} \end{array} \end{array}$$

(8) $$\begin{array}{l} \text{RE (\%) = (((}\sum_{\text{n=1}}^{\text{n}}{{\text{(E}}_{\text{i}}{\text{ - O}}_{\text{i}}\text{)}}^{\text{2}}{\text{) / n)}}^{\text{1/2}}{\text{)/ O}}_{\text{i}}^{\prime }\text{,} \end{array}$$

Where *E*_*i*_ and *O*_*i*_ are the estimated and the observed values of AGB, PNU, or NNI, respectively. *O'*_*i*_ is the mean of the observed values of the AGB, PNU, or NNI. *n* is the number of samples.

To compare these two strategies, relative R^2^ (RR^2^), relative RMSE (RRMSE), relative RE (RRE), and the relative number of variables (RN) were calculated by normalizing them using the maximum values of R^2^, RMSE, RE and the number of variables, respectively.

#### Identifying Appropriate Combinations of Multi-Source Data for N Status Estimation

To determine the effects of different sources of data on N status estimation, distinct combinations of multi-source data were explored, which consisted of (1) only NDRE; (2) both NDRE and climatic data (NDRE+C); (3) both NDRE and management data (NDRE+M), and (4) full sources of data (NDRE+C+M). The models with NDRE only serve as a reference for further assessment of the benefit of adding climatic and/or management data. The decrease of RMSE (dRMSE) was defined as the ratio of the RMSE difference obtained from the multi-source data (RMSE_multi−source_) and NDRE only (RMSE_NDRE_) to the RMSE_NDRE_. This was calculated as follows:


$$\begin{array}{l} {\text{dRMSE (\%) = (RMSE}}_{\text{multi-source}}{\text{ − RMSE}}_{\text{NDRE}}\text{)}\\ {\text{ /RMSE}}_{\text{NDRE}} \end{array}$$

where *RMSE*_*NDRE*_ and *RMSE*_*multi*−*source*_ are RMSE of NDRE only and combinations of multi-source data, respectively.

#### Modeling Process

Microsoft Excel (Microsoft Corporation, Redmond, Washington, USA) was used to establish and select SLR models with the highest R^2^. The Scikit-learn library (in Python 3.9.5) was utilized to establish the RFR models (Abraham et al., 2014). Three hyper-parameters, namely, the number of decision trees (n_estimators, from 10 to 300 at intervals of 50), the maximum depth (max_depth, from 2 to 20 at intervals of 2), and the minimum number of samples to spilt (min_samples_split, from 2 to 12 at intervals of 2) were tuned using 5-fold cross-validated grid search (Abraham et al., 2014).

#### Evaluating Strategies and Performance Metrics

The total data of AZ1, AZ2, and AZ1+2 were split randomly into 70% for training and 30% for the test. Meanwhile, 5-fold cross-validation was implemented due to its simplicity, universality, and efficiency in reducing the over-fitting problem (Arlot and Celisse, 2010), and its mean values were used to represent the predictive performance. The accuracy of trained models was evaluated using the test dataset with R^2^, RMSE, and RE.

## Results

### Variability of Winter Wheat N Status Indicators

A total of 396 observations were obtained, with 277 for training and 119 for the test (Table 2). PNU showed the highest variation on training dataset (CV = 60.53, 51.04 and 52.29% in AZ1, AZ2 and AZ1+2, respectively), followed by AGB (CV = 50.2, 38.53, and 41.56% in AZ1, AZ2 and AZ1+2, respectively) and NNI (33.67, 34.75, and 32.35% in AZ1, AZ2, and AZ1+2, respectively). Similar results were presented in the test dataset. In general, experimental data is suitable to evaluate the predictive performance for N status estimation.

**Table 2:** Descriptive statistics of winter wheat aboveground biomass (AGB), plant N uptake (PNU), and N nutrition index (NNI) for training and test dataset in different agro-ecological zones.

	**Agro-ecological zone 1**					**Agro-ecological zone 2**					**Agro-ecological zone 1+2**				
	**Min**	**Max**	**Mean**	**SD**	**CV(%)**	**Min**	**Max**	**Mean**	**SD**	**CV(%)**	**Min**	**Max**	**Mean**	**SD**	**CV(%)**
**Training dataset**	(*n =* 88)					(*n =* 189)					(*n =* 277)				
AGB (t ha^−1^)	0.83	6.04	2.79	1.40	50.20	0.81	5.77	2.66	1.03	38.53	0.56	5.68	2.63	1.09	41.56
PNU (kg ha^−1^)	15.82	233.63	86.33	52.25	60.53	17.87	190.35	79.34	40.45	51.04	12.50	233.63	79.68	41.66	52.29
NNI	0.42	1.92	1.05	0.35	33.67	0.28	1.87	1.03	0.36	34.75	0.42	1.92	1.04	0.34	32.35
**Test dataset**	(*n =* 38)					(*n =* 81)					(*n =* 119)				
AGB (t ha^−1^)	0.56	5.40	2.77	1.19	42.88	0.58	5.85	2.47	1.13	45.74	0.81	6.04	2.74	1.29	47.46
PNU (kg ha^−1^)	12.50	190.39	87.67	46.75	53.33	15.40	224.69	71.70	41.52	57.91	17.87	224.69	81.18	50.16	61.78
NNI	0.43	1.64	1.08	0.36	33.60	0.36	1.96	0.97	0.32	33.04	0.28	1.96	1.01	0.38	38.24

### Estimating N Status Indicators Using Simple Linear Regression Models

**Figure 3:**
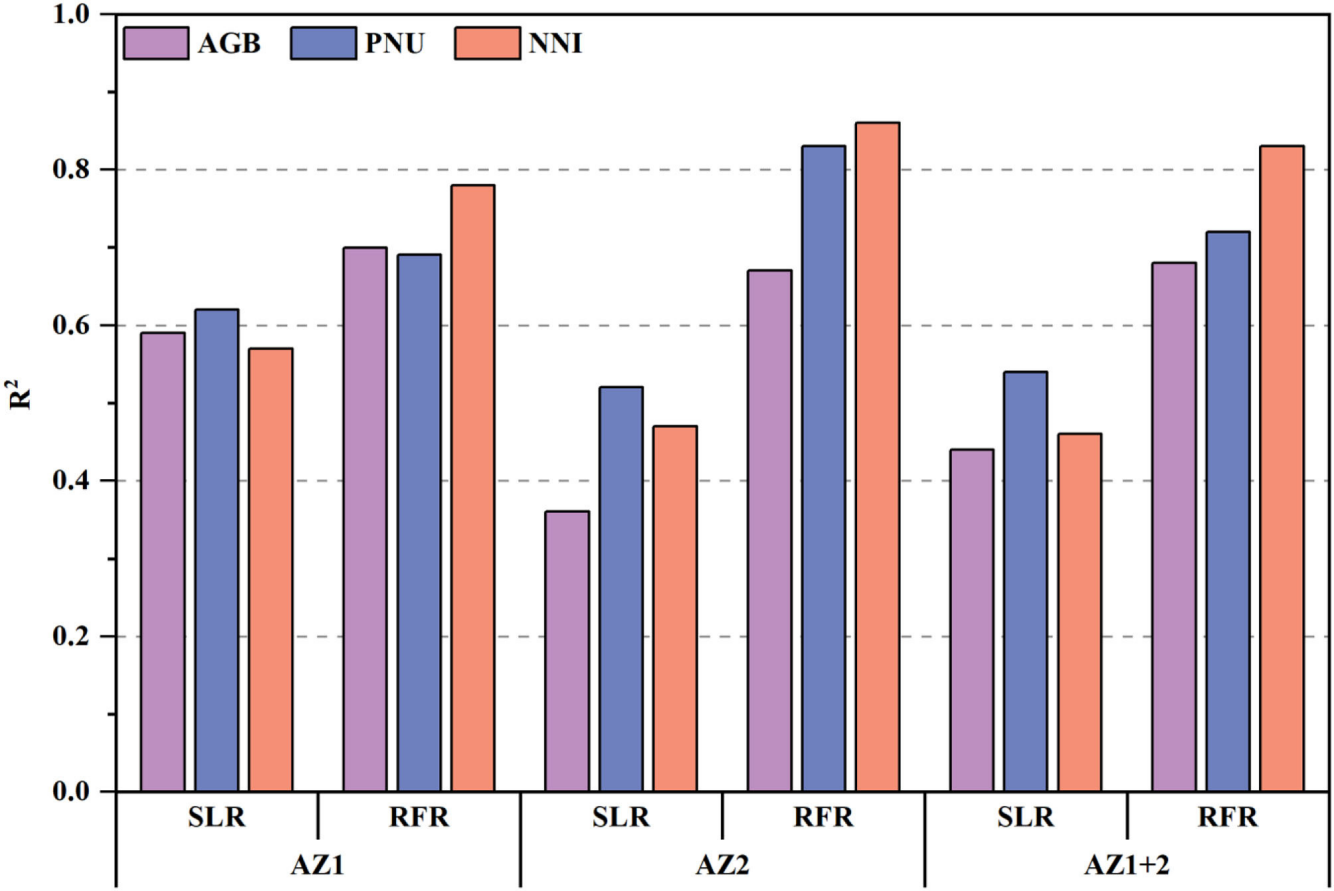
Coefficient of determination (R^2^) of simple linear regression (SLR) and random forest regression (RFR) for estimating aboveground biomass (AGB), plant N uptake (PNU) and N nutrition index (NNI) in agro-ecological zone 1 (AZ1), agro-ecological zone 2 (AZ2) and across two agro-ecological zones (AZ1+2) on the training dataset, respectively.

**Figure 4:**
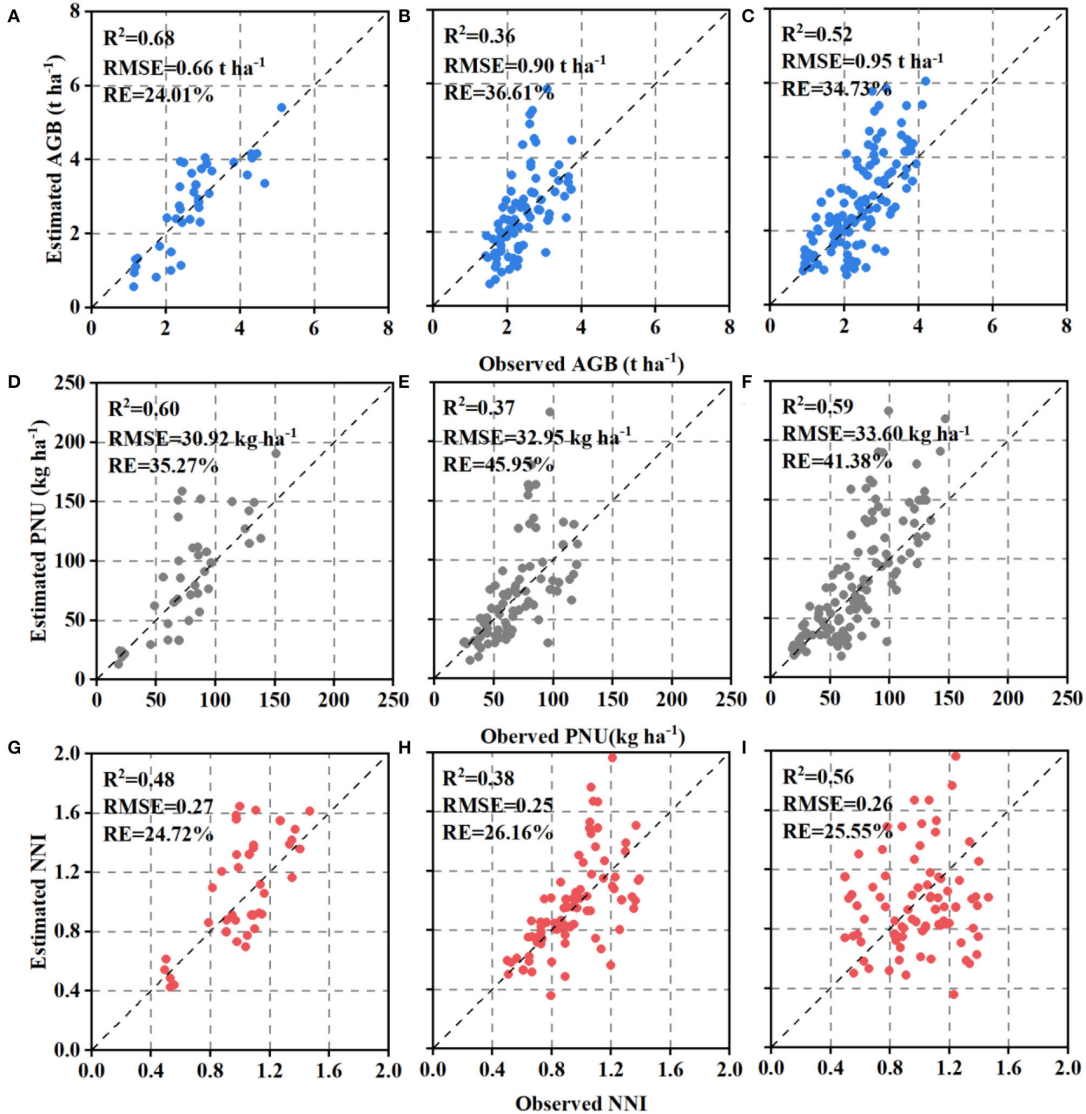
Relationships between estimated and observed aboveground biomass (AGB), plant N uptake (PNU), and N nutrition index (NNI) using simple linear regression on the test dataset in agro-ecological zone 1 **(A,D,G)**, agro-ecological zone 2 **(B,E,H)** and two agro-ecological zones **(C,F,I)**. The black dotted line is the 1:1 line.

The performance of SLR models for estimating N status indicators varied slightly across the different AZs (Figure 3). Comparatively, NDRE explained 59% of AGB variation, 62% of PNU, and 57% of NNI in AZ1, which performed better than AZ2 with 36, 52, and 47%, respectively. The performance of models in AZ1+2 was similar to that in AZ2 (44% for AGB, 54% for PNU, and 46% for NNI). The validation results confirmed that models for AZ1 (Figures 4A,D,G) had lower or similar RMSE and RE than models for AZ2 (Figures 4B,E,H) or AZ1+2 (Figures 4C,F,I), respectively.

### Selection of Important Variables

**Figure 5:**
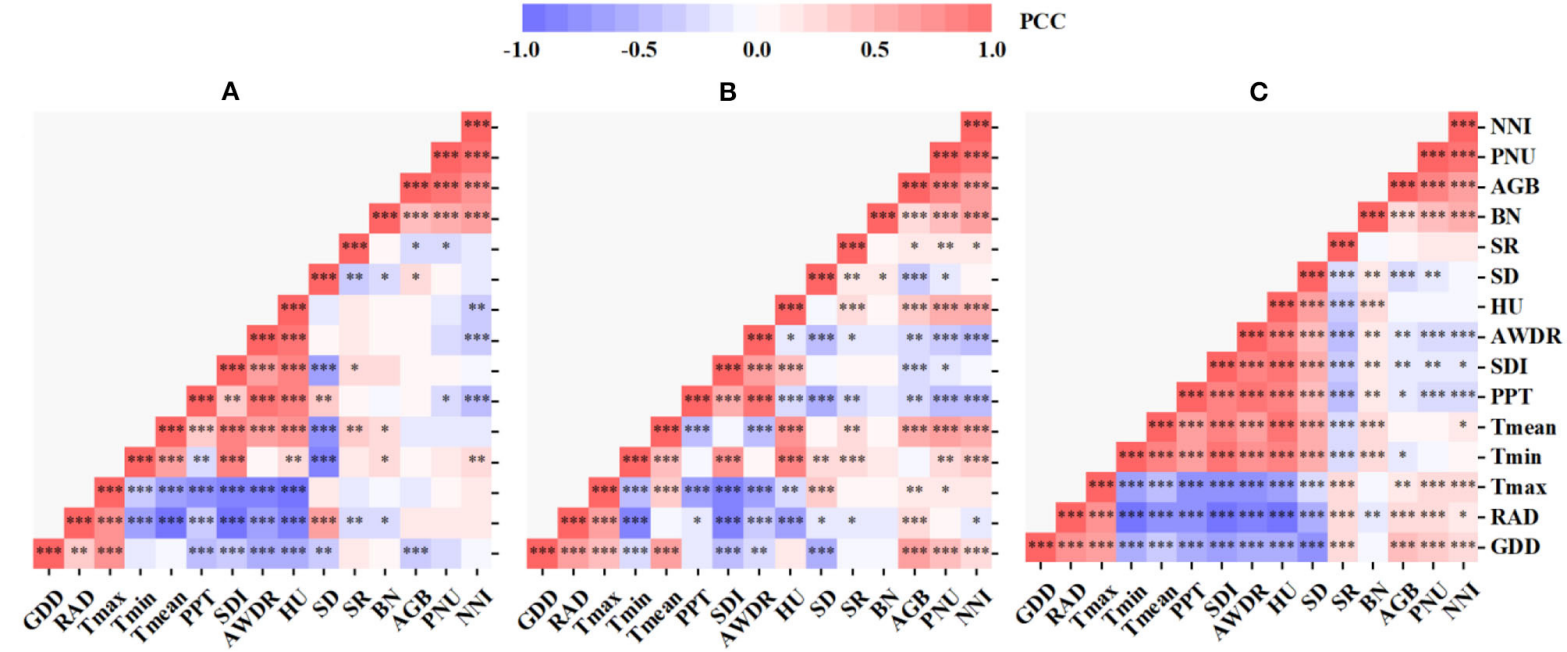
Pearson correlation coefficient (PCC) analysis in agro-ecological zone 1 **(A)**, agro-ecological zone 2 **(B)** and across two agro-ecological zones **(C)**. “***”, “**”, and “*” note the significant correlation at 0.001, 0.01, and 0.05, respectively. GDD, RAD, T_max_, T_min_, T_mean_, PPT, SDI, AWDR, HU, SD, SR, BN, AGB, PNU, and NNI refer to growing degree days, solar radiation, seasonal maximum, minimum, and mean temperature, seasonal total precipitation, shannon diversity index, abundant and well-distributed rainfall, relative humidity, sowing dates, seeding rates, basal N, aboveground biomass, plant N uptake, and N nutrition index, respectively.

For the PCC, the correlation and corresponding p-values were analyzed between candidate independent variables and dependent variables. Notably, there were both positive and negative correlations in each group (Figure 5). For the temperature-related variables, GDD was selected first due to its highest correlation with AGB in AZ1, and RAD was selected for its high correlation with AGB but lower correlation with GDD (<0.5). Similarly, PPT and SDI were selected from the water-related variables, while BN and SR from the management variables (Figure 5A). The above steps were repeated for AGB, PNU, and NNI in AZ2 (Figure 5B) and AZ1+2 (Figure 5C), respectively. Overall, six variables were selected in both AZ1 and AZ2 and five variables in AZ1+2.

**Figure 6:**
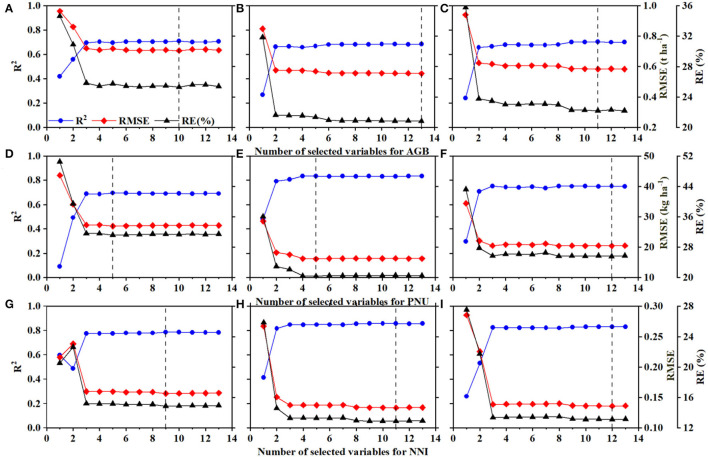
The accuracy of aboveground biomass (AGB), plant N uptake (PNU), and N nutrition index (NNI) with variables selected by the random forest regression in agro-ecological zone 1 **(A,D,G)**, agro-ecological zone 2 **(B,E,H)** and across two agro-ecological zones **(C,F,I)**.

For the VIRRFR, cross-validation accuracy improved significantly within the top two or three variables and then fluctuated slightly (Figure 6). Variables were determined by taking the highest R^2^, lowest RMSE, and RE into account. For example, when the AGB model achieved the largest R^2^ (0.71), the lowest RMSE (0.71 t ha^−1^) and RE (25.3%) in AZ1, the 10 variables in the models were selected as the optimal variables (Figure 6A). In the same way, the number of selected variables for AGB (13 in AZ2 and 11 in AZ1+2, Figures 6B,C), PNU (5 in AZ1, AZ2, and 12 in AZ1+2, Figures 6D–F), and NNI (9 in AZ1, 11 in AZ2, and 12 in AZ1+2, Figures 6G–I) were determined.

### Estimating N Status Indicators by Random Forest Regression Models

#### Models Construction

**Figure 7:**
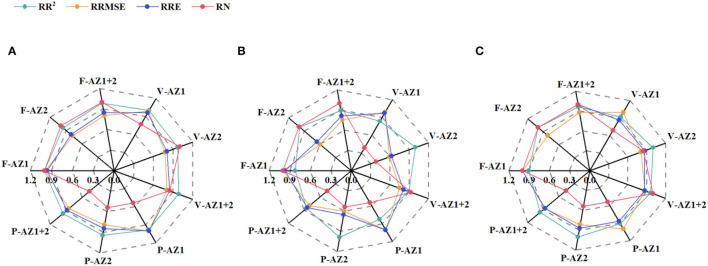
The accuracy of random forest regression (RFR) on aboveground biomass **(A)**, plant N uptake **(B)**, and N nutrition index **(C)** using two variable selection strategies and full variables. F-AZ1 represents full variables in agro-ecological zone 1, P-AZ1 represents selected variables by Pearson correlation coefficient strategy in agro-ecological zone 1, V-AZ1 represents selected variables by the variable importance ranked by the RFR strategy in agro-ecological zone 1; similarly, F-AZ2, P-AZ2, V-AZ2, F-AZ1+2, P-AZ1+2, V-AZ1+2 represents full variables, selected variables by Pearson correlation coefficient strategy and the variable importance ranked by the RFR strategy in agro-ecological zone 2 and across two agro-ecological zones, respectively. RR^2^, RRMSE, RRE, and RN represent the relative coefficient of determination, relative root mean square error, relative error, and the relative number of variables, respectively.

The RFR models were trained using full variables and selected variables based on the above-mentioned strategies and then were evaluated to determine the optimal models based on cross-validation (Figure 7). Slight differences in R^2^, RMSE, and RE were observed between the models with variable selection and full variables, while PCC showed the superiority due to the least variables with the same accuracy except for the PNU estimation models in AZ1 and AZ2. Thus, the variables selected by the PCC were considered optimal and analyzed in the next part. It should also be pointed out that similar performance of models was shown in AZ1+2 compared to AZ1 and AZ2, indicating the consistency of constructed models.

The performance of RFR models for N status estimation was consistently better than SLR models. All the R^2^ values of RFR models were significantly higher than the corresponding R^2^ values of SLR models, and the most obvious improvement appeared in AZ2, followed by AZ1 (Figure 3). Consistently, the AZ1+2 matched the above result. In terms of N status indicators, the NNI estimation models performed the best (R^2^ = 0.78–0.83), followed by PNU (R^2^ = 0.69–0.83) and AGB (R^2^ =0.67–0.70).

#### Models Evaluation

**Figure 8:**
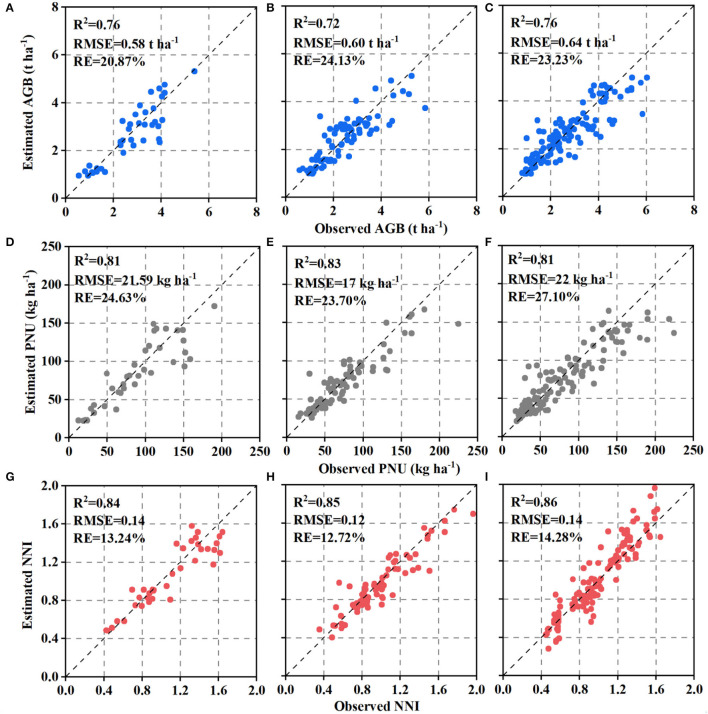
The performance of random forest regression using the variables selected from the Pearson correlation coefficient strategy to predict the aboveground biomass (AGB), plant N uptake (PNU) and N nutrition index (NNI) in agro-ecological zones 1 **(A,D,G)**, agro-ecological zone 2 **(B,E,H)** and two agro-ecological zones **(C,F,I)**. The black dotted line is the 1:1 line.

In general, the observed N status indicators were more related to the estimated ones obtained from RFR (Figure 8) than SLR models (Figure 4). Moreover, the improvement of RFR models over SLR models was larger in AZ2 than in AZ1 (Figures 8A,B,D,E,G,H). On the other hand, the validation results indicated the estimation accuracy varied with N status indicators, with NNI having the best result (Figures 8G–I).

### Importance of Different Variables

**Figure 9:**
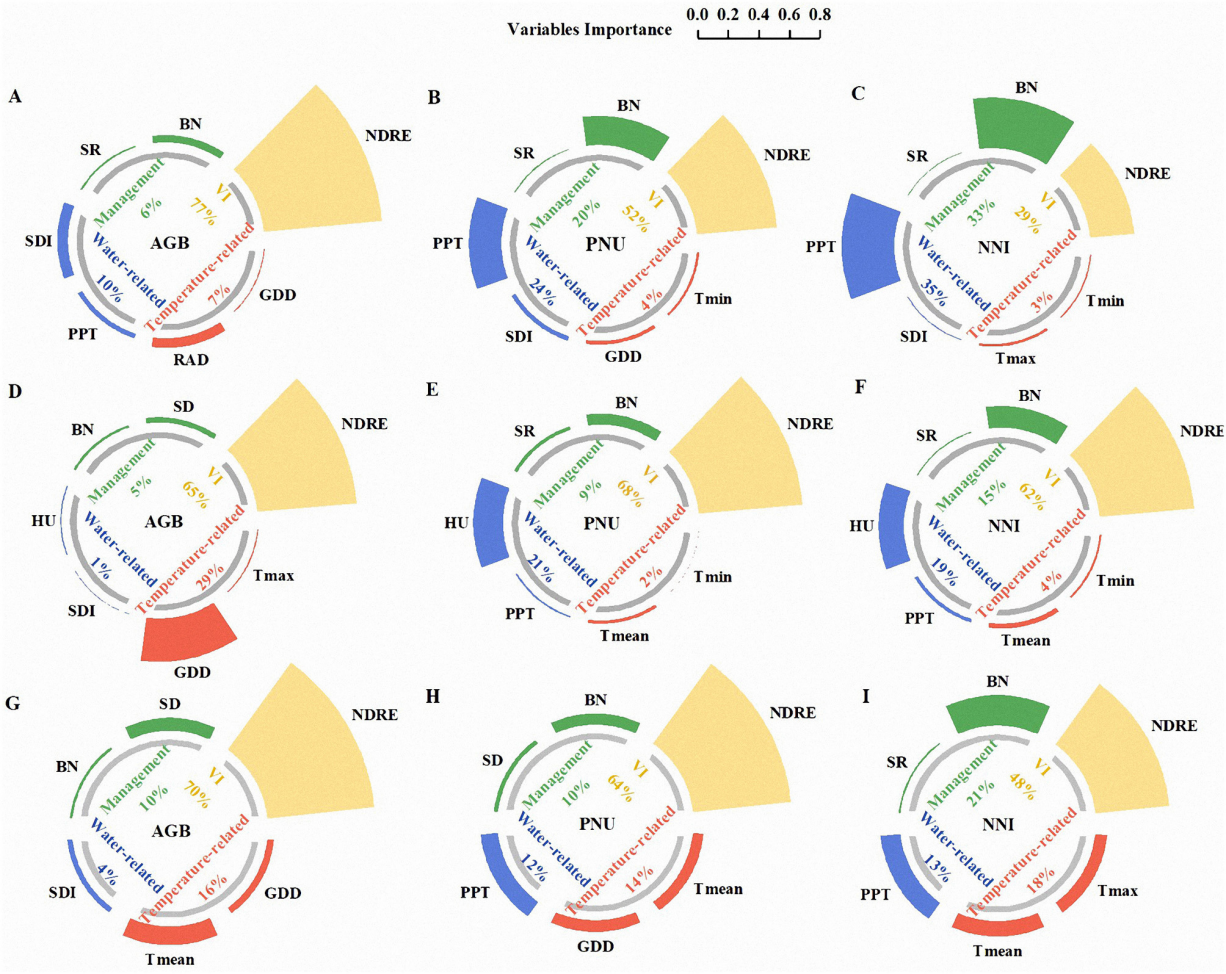
The importance of variables of NDRE, climatic, and management factors for estimating the aboveground biomass, plant N uptake, and N nutrition index in agro-ecological zone 1 **(A–C)**, agro-ecological zone 2 **(D–F)**, and across two agro-ecological zones **(G–I)**. GDD, RAD, T_max_, T_min_, T_mean_, PPT, SDI, HU, SD, SR, BN, AGB, PNU, and NNI refer to growing degree days, solar radiation, seasonal maximum, minimum, and mean temperature, seasonal total precipitation, shannon diversity index, relative humidity, sowing dates, seeding rates, basal N, aboveground biomass, plant N uptake, and N nutrition index, respectively.

The importance of variables selected by the PCC for estimating N status indicators was explored by the RFR models (Figure 9). The results showed that NDRE was consistently viewed as the most important variable except for the estimation of NNI in AZ1, and BN was considered important for all N status indicators. It should be noted that the important variables differed with the AZs and N status indicators. In AZ1, SDI and RAD were ranked as the top two variables for AGB, PPT and BN for PNU, and PPT and BN for NNI (except for NDRE). The water-related variables were consistently identified to be the vital variables (Figures 9A–C). Compared to AZ1, temperature-related variables were also of great importance to AZ2 (Figure 9D). Generally, water and temperature-related variables were listed as the most critical variables across AZs (Figures 9G–I). With respect to N status indicators, temperature-related variables were more important for AGB (Figures 9D,G), while water-related variables were more important for PNU and NNI (Figures 9B,C,E,F).

### Multi-Source Data Performance

**Figure 10:**
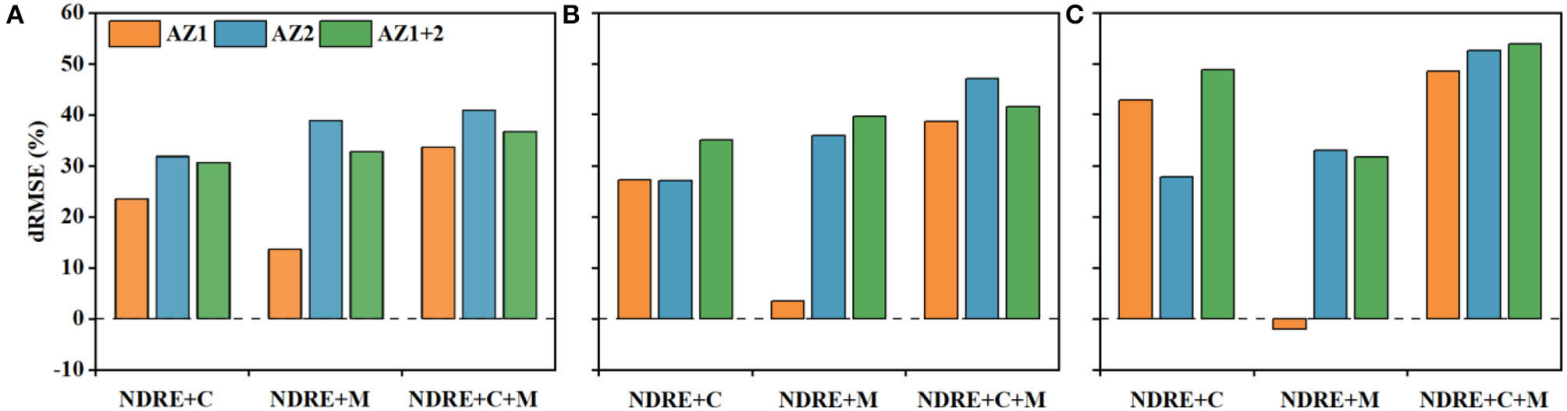
The performance of the decrease of RMSE (dRMSE, %) of multi-source data for the aboveground biomass **(A)**, plant N uptake **(B)**, and N nutrition index **(C)** by combining climatic (C) factors and management (M) factors compared to normalized difference red edge (NDRE) only in agro-ecological zone 1 (AZ1), agro-ecological zone 2 (AZ2) and across two agro-ecological zones (AZ1+2).

Different combinations of variables were further investigated and evaluated for the estimation of N status indicators based on dRMSE. The results indicated that the more the sources, the better the model's performance (Figure 10). Specifically, the dRMSE of two-source data (NDRE+C or NDRE+M) was more significant than that of single-source data (NDRE), and full sources of data achieved the best results, with 33.72–40.95% for AGB, 38.56–46.99% for PNU, and 48.54–52.50% for NNI. The combination of NDRE+C produced better results than those of NDRE+M (23.55, 27.21, and 42.83% vs. 13.62, 3.42, and −2.1% for AGB, PNU, and NNI, respectively), indicating that climatic data make more important contributions to the estimation of N status indicators in AZ1. On the contrary, the combination of NDRE+M performed better than NDRE+C in AZ2. Adding the climatic variables produced the greater dRMSE for AGB in AZ2 than that in AZ1, while the opposite was true for NNI. For PNU, the performances of adding the climatic variables were similar in AZ1 and AZ2. In AZ1+2, the contributions of the climatic data to NNI were greater than PNU.

## Discussion

### Comparison of Different Regression Models

The SLR models are commonly used to estimate plant N status and guide variable N applications (Li et al., 2019; Wang et al., 2019). However, models using NDRE could only explain 48–68% of N status indicators variabilities for AZ1, 36–38% for AZ2, and 52–59% for AZ1+2 based on the test dataset (Figure 4). Those results agreed with the findings of wheat N status estimation only using VIs across farmer's fields in a village (Chen et al., 2019). The performance difference of these models between AZ1 and AZ2 may be attributed to the different experimental treatments (Table 1) and climatic and management factors (Figure 1B). Consequently, it is necessary to combine climatic and management factors with NDRE in RFR models to improve the estimation accuracy. In agreement with Cummings et al. (2021) and Wang et al. (2021), the multi-variable RFR models performed better than SLR models (Figure 3), because the RFR models included multi-source data and could analyze nonlinear and complex relationships (Wang et al., 2021).

### Comparison of Variable Selection Strategies

Generally, variable selection prior to model construction could reduce the data dimension and measurement while differentiating meaningful variables from the noise and improving calibration efficiency (Heremans et al., 2015; Feng et al., 2020). Two variable selection strategies, the PCC and VIRRFR, were adopted in this study. In Figure 7, the performance of full variables models was not better than those of using fewer variables with variable selection, demonstrating the effectiveness of variable selection. Similar results were observed in the previous studies (Cai et al., 2019; Zhang et al., 2019; Wang et al., 2020). The PCC showed more potential than VIRRFR to estimate N status, except for the estimation of PNU in AZ1 and AZ2. The variables were selected based on the PCC strategy from a statistical analysis point of view, resulting in more informative and diverse variables. Many variables were correlated with each other at the p < 0.001 significance level but still selected in the VIRRFR strategy (data not shown), such as T_max_ and T_mean_ of AZ1 (Figure 5).

### Comparison of Uncertainties for Two Agro-Ecological Zones

Consistent with prior studies, NDRE, a useful proxy reflecting plant growth and development, has been deemed the most important variable for crop N status estimation (Osco et al., 2020; Colaço et al., 2021). Nonetheless, it is climatic and management factors that affect winter wheat growth and development. Accordingly, this study focused on the values of climatic and management factors for wheat N status estimation in different AZs. It is not surprising that these factors had different contributions in different AZs and for different N status indicators. Water-related variables played a more important role in AZ1, while both water-related and temperature-related variables were important in AZ2 (Figures 9D–F). Indeed, unlike AZ1, with a dry climate and sufficient sunshine, AZ2 belongs to a subtropical monsoon climate and is characterized by abundant precipitation and relatively less sunshine. In addition, the contributions of temperature-related variables to improve the estimation accuracy of AGB were greater than those of water-related variables, which were more correlated to PNU and NNI. A possible reason is that temperature has a greater effect on crop yield that is determined by the accumulation and redistribution of dry matter (Lee and Tollenaar, 2007) than rainfall (Schlenker and Lobell, 2010), because the dry matter of plants is formed by intercepting solar radiation and physiological and biochemical processes (Xue et al., 2002). Another possible reason is that the crop root system influenced by the water directly or indirectly is critical to absorbing nutrients from the soil, like N (Walsh et al., 2012; Sharma et al., 2018).

Overall, the results substantiated the viewpoint that integrating more sources of data will lead to better estimation performance (Chen et al., 2021; Gua et al., 2021; Liu et al., 2021; Li et al., 2022). Regarding their combinations, the results varied with sites and N status indicators. As for AGB, adding climatic factors improved the model performance more in AZ2 than in AZ1. Metabolic imbalances induced by lower average temperature during the vegetative stages could retard crop germination and plant growth (Ye et al., 2020). The difference of phenological information (SE stage, Table 1) would change AGB accumulation because it could, directly and indirectly, influence photosynthesis and respiration (Gua et al., 2021). On the contrary, adding the climatic factors improved the estimation of NNI in AZ1 more than AZ2, which could be caused by the influence of the length of the growth period or the responses of winter wheat cultivars to climate variability (Tao et al., 2014). In AZ1+2, the contributions of the management factors to PNU were greater than climatic factors because PNU showed a positive correlation to N rates (Egan et al., 2019; Sandaña et al., 2021). The sowing date in AZ2 was later than that in AZ1, leading to lower AGB accumulation, the number of tillers and plant height due to low temperature in the vegetative stages and higher temperature in the reproductive stages (Fazily, 2021). In contrast, a suitable sowing date can make full use of natural resources, such as light, heat, and water, enhancing plant population and facilitating the accumulation and uptake of N before winter. Different variables could interact with each other to influence crop growth and N status. For example, higher seeding rates could make up for stagnant tiller development, which would benefit cultivars with fewer tillers (Staggenborg et al., 2003).

### Applications and Future Work

In this study, the estimation models across two AZs showed similar results to that of a single AZ. Remarkably, it demonstrated the robustness and application of the established RFR approach.

While the results of this study were encouraging, there is still room for improvement. A total of 13 variables were considered in this study, which may be insufficient for constructing a reliable model for wider applications due to limitations in data amount and quality, model representativeness, and the number of key variables (Chlingaryan et al., 2018). Although the active canopy sensors are promising for evaluating N status, as indicated in this study, unmanned aerial vehicles and satellite remote sensing are more efficient for larger regions or farms (Huang et al., 2017; Chen et al., 2019). In this regard, follow-up research is needed to expand the scale of the study regions, supplement more climatic and management factors that directly or indirectly influence winter wheat growth, and develop more applicable N estimation models using unmanned aerial vehicles or satellite remote sensing images.

## Conclusion

The present work explored the adaptation and feasibility of RFR integrating NDRE, climatic, and management factors for N status estimation compared with SLR using only NDRE. The results indicated that RFR yielded better stability and higher accuracy. Variable selection was essential to construct effective models using fewer variables to achieve similar results compared to models using full variables, and PCC was confirmed to be an effective approach. Besides, dominant variables varied with AZs and N status indicators. At higher latitudes, climatic factors were more important to N status estimation, especially water-related factors. In addition, climatic factors significantly improved the performance of NNI estimation. Although climatic factors were crucial to AGB estimation, management factors were more important for N status estimation at lower latitudes. More studies are needed to develop unmanned aerial vehicles and satellite remote sensing-based ML models incorporating multi-source data for more efficient monitoring of crop N status under more diverse soil, climatic, and management conditions across large regions.

## Data Availability Statement

The original contributions presented in the study are included in the article/supplementary material, further inquiries can be directed to the corresponding author.

## Author Contributions

QC, WC, and YM conceived and designed the experiments. YL and QC performed the experiments, analyzed the data, and wrote the original manuscript. QC, YM, DC, JZ, and SL reviewed and revised the manuscript. All authors read and approved the final manuscript.

## Funding

This research was funded by the Jiangsu Province Key Technologies Research and Development Program (BE2021308), the National Natural Science Foundation of China (31601222), the Norwegian Ministry of Foreign Affairs (SINOGRAIN II, CHN-17/0019), the Academic Program Development of Jiangsu Higher Education Institutions (PAPD), and the 111 Project (B16026).

## Conflict of Interest

The authors declare that the research was conducted in the absence of any commercial or financial relationships that could be construed as a potential conflict of interest.

## Publisher's Note

All claims expressed in this article are solely those of the authors and do not necessarily represent those of their affiliated organizations, or those of the publisher, the editors and the reviewers. Any product that may be evaluated in this article, or claim that may be made by its manufacturer, is not guaranteed or endorsed by the publisher.

## Appendix

**Table A1:** Symbols and acronyms used in the article.

AGB	aboveground biomass
AWDR	abundant and well-distributed rainfall
AZ1	agro-ecological zone 1
AZ1+2	two agro-ecological zones
AZ2	agro-ecological zone 2
BN	basal N
D_max_	daily maximum temperature
D_min_	daily minimum temperature
dRMSE	the decrease of RMSE
FM	farmer's management
GDD	growing degree days
HU	relative humidity
N	Nitrogen
N_c_	critical N concentration
NDRE	normalized difference red edge
NDRE+C	both NDRE and climatic data
NDRE+C+M	full sources of data
NDRE+M	both NDRE and management data
NNI	N nutrition index
PCC	Pearson correlation coefficient
PNM	precision N management
PNU	plant N uptake
PPT	seasonal total precipitation
RAD	solar radiation
RFR	random forest regression
RN	the relative number of variables
RONM	regional optimum N management
RR^2^	relative R^2^
RRE	relative RE
RRMSE	relative RMSE
SDI	shannon diversity index
SE	stem elongation
SLR	simple linear regression
T_base_	base temperatures
T_max_	seasonal maximum temperature
T_mean_	seasonal mean temperature
T_min_	seasonal minimum temperature
VIRRFR	the variable importance ranked by the RFR
VIs	vegetation indices
